# Coaxial Spring-Like Stretchable Triboelectric Nanogenerator Toward Personal Healthcare Monitoring

**DOI:** 10.3389/fbioe.2022.889364

**Published:** 2022-04-13

**Authors:** Jinmei Liu, Saixuan Li, Maosen Yang, Yuxin Wang, Nuanyang Cui, Long Gu

**Affiliations:** School of Advanced Materials and Nanotechnology, Xidian University, Xi’an, China

**Keywords:** stretchable triboelectric nanogenerator, biomechanical energy harvesting, coaxial fiber structure, gesture monitoring, self-powered temperature sensor

## Abstract

Stretchable triboelectric nanogenerators have attracted increasing interests in the field of Internet of Things and sensor network. Therefore, great efforts have been made to realize the stretchability of electronic devices *via* elaborated material configurations and ingenious device designs. In this work, a flexible and stretchable TENG is developed with a coaxial spring-like structure. The unique structure allows it to generate electrical energy for different degrees of stretching deformations. Its output demonstrates good response to the strain and frequency of the mechanical deformation. At the same time, it exhibits excellent stability and washability. The TENG can be worn on the human fingers, elbow, and knee to monitor the body activities. Furthermore, a self-powered temperature sensor system is fabricated by integrating the TENG with a temperature sensor to identify the operating ambient temperature in real time. A combination of this flexible and stretchable TENG with body motions and a temperature sensor brings a novel insight into wearable functional electronics and user-friendly health monitoring, which has an important basic research significance and practical application value in biometric systems.

## Introduction

Due to the rapid advances in electronics, computing, and integration technologies over the past decade, electronic devices have been trending toward becoming lighter, thinner, smaller, and softer. Since 2012, triboelectric nanogenerators (TENGs) have been developing with the ability of harvesting mechanical energies from the working environment and converting them into electrical energy based on coupling between triboelectrification and electrostatic induction effect (Fan et al., 2012; Zhu et al., 2012; Wang and Wang, 2019; Zhang et al., 2020a; Zhang et al., 2021). With both excellent energy conversion efficiency and possibility of using diverse materials, TENGs have been utilized in a wide range of applications such as wearable electronics, human–machine interfaces, and self-powered sensors (Lai et al., 2016; Li et al., 2016; Park et al., 2016; Bai et al., 2018; Wu et al., 2019; Zhang et al., 2020b; Li et al., 2021). The wearable TENGs can be attached or even wore on the human body to implement the biomedical health monitoring and human motions sensing through harvesting energy generated by human motions, such as walking, running, jumping, tapping, and swinging or bending the arm (Cui et al., 2015; Niu et al., 2015; Pu et al., 2016; Liu et al., 2019; Qi et al., 2020; Cheng et al., 2021). However, intimately involved in the human-body movement response, the wearable TENGs lack high-performance elasticity to properly self-expand and self-loosen. Therefore, flexible and stretchable TENGs are being one of the research hotpots to develop their potential applications in healthcare, energy, and military purposes (Lin et al., 2017; Parida et al., 2019a; Sheng et al., 2021).

Generally, a TENG is composed of three parts: the triboelectric layers to generate triboelectric charges, electrodes to extract charges, and the spacer to separate the triboelectric layers. So, to prepare the stretchable TENGs, a lot of work focused on embedding electronic conductors, such as conductive polymers, silver nanowires, carbon black/nanotubes, graphite spray, and liquid metals, into extendible matrixes to provide the elasticity as the electrode materials and electrification materials (Wang et al., 2016; Dickey, 2017; Yang et al., 2018; Parida et al., 2019b; Han et al., 2020). Under local deformation and stretching condition, these soft conductive composite materials could effectively respond to the external force, but it seems to be powerless under large deformation with irreversible damage due to uneven mixing and poor compatibility (Fang et al., 2016; Mu et al., 2016; Guo et al., 2017). Relatively speaking, the ionic conductor transfers charges through ions, which can be transported using deformable soft polymeric materials. Consequently, the stretchable and conductive hydrogel electrolyte attracted a lot of attentions with its capacity of high flexibility and high elasticity (Fang et al., 2016; Pu et al., 2017; Wang et al., 2018; Sun et al., 2019; Wang et al., 2020a; Wang et al., 2020b; Kim et al., 2020). However, in most of the reported hydrogel-based TENGs, excellent stretching, mechanical toughness, and electrical conductivity cannot be achieved at the same time due to the dehydration or evaporation of the liquid solvent, which largely limits their applications (Niu et al., 2013; Xu et al., 2013; Shuai et al., 2020). In addition to thinking from the materials’ point of view, researchers also worked hard to develop new manufacturing processes to design stretchable and deformable TENGs that integrate the structure and function simultaneously. With innate tensile properties, traditional origami and kirigami patterns have been used to fabricate the stretchable TENG, which has provided a structure design strategy using a simple and mature processing technology to make the essentially inelastic material to be available for TENGs. However, their stretchability was quite limited compared with other methods, and Young’s modulus of the paper is quite high and easily damaged by reciprocating stretching, resulting in unstable output performance (Yang et al., 2015; Guo et al., 2016; Wu et al., 2016; Lu et al., 2018).In addition, traditional sewing technologies based on textiles and fabrics, such as weaving, knitting, serpentine sewing, and spiral winding, also have advantages in texturing the stretchable structures. Thus, many kinds of two-dimensional (2D) and three-dimensional (3D) orthogonally woven TENGs have been developed to be flexible, stretchable, and comfortable using fabric fibers and conductive fibers (Kim et al., 2015; Zhao et al., 2016; Dong et al., 2017; Gong et al., 2019; Zhu et al., 2019; Cong et al., 2020). Although these TENGs are flexible and stretchable to some extent, the inherent strain constraints between the electrode materials and the electrification materials prevent them from achieving the maximum stretchability. To investigate the fundamental problems, the difficulty lies in the stretchable TENG’s working requirements to endure deformation not only in each component but also in the entire TENGs. Therefore, to design a geometric structural with high stretchability, structural integrity, and conformability plays an important role in flexible and stretchable TENGs research imposed by the increasing application requirements.

Here, we introduce a stretchable TENG with a coaxial spring-like structure *via* a simple and effective route, which could be utilized in personal healthcare monitoring. This TENG has excellent flexibility and stretchability and can be folded and bended into different shapes and stretched to different lengths, while it has good stability and washability. It can generate electrical energy under different external mechanical deformation. When worn on the four fingers, it could identify the finger motions. Furthermore, when worn on the elbow and knee, it could respond to the arm and leg activities. At the same time, an output voltage of 0.6 and 1.7 V, and an output current of 10.6 and 25.0 nA can be detected, respectively. Moreover, a temperature sensor is employed to build a self-powered system with the TENG, which could successfully identify the temperature under the working condition. This work may not only promote human biomechanical energy harvesting but also provide a novel design concept of the fiber-based TENG and expand their scope for wearable electronics application in the era of IoT.

## Results and Discussion

**FIGURE 1:**
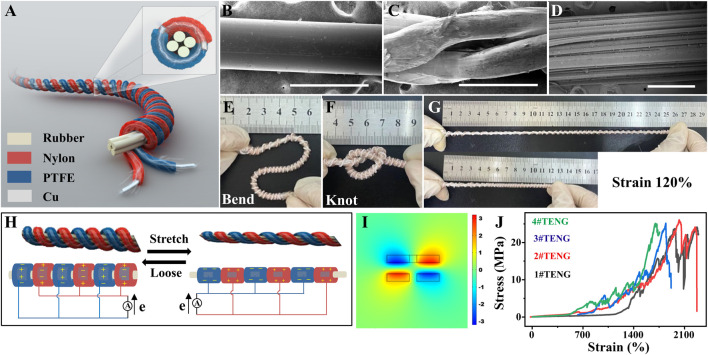
Structure and working mechanism of the TENG. **(A)** Structural diagram of the TENG. **(B–D)** SEM images of the rubber fiber, the nylon fiber, and the PTFE fiber, respectively. Scalebar in **(B–D)** is 500 μm. **(E–G)** Photograph of the flexible and stretchable TENG in different shapes. **(H)** Working principle of the TENG. **(I)** Numerical calculation of the corresponding electrical potential distribution by COMSOL software. **(J)** Stress–strain curve of the four identical TENGs.

Figure 1A is the structural diagram of the TENG in a multi-dimensional core–shell structure. At first, an inner core is designed using four rubber fibers, which is made up of many fine fibers as shown in Figure 1B. Then, two kinds of composite fibers in the core–shell structure are enwound around the rubber fibers side by side. As for the two composite fibers, one is a nylon-coated copper wire, and another is a PTFE-coated enameled copper wire, whose detailed experimental process can be referred to our previous work (Liu et al., 2019). The two copper wires in the two composite fibers act as the positive and negative electrodes, respectively. The surface topography of the nylon fiber and the PTFE fiber that we used are observed by SEM as shown in Figures 1C,D. In this way, the elastic core substrate structure and the helical surrounding triboelectric layers structure together form the spring-like coaxial energy fiber. As demonstrated in Figures 1E–G, it can be bent into various shapes, knotted, and stretched, exhibiting excellent flexibility and stretchability, which makes it adaptable to irregular surfaces and appropriate in different human body motion situations. The operating principle of the TENG is briefly described in Figure 1H. As we all know, the electron affinity of the PTFE is higher than that of the rubber. Meanwhile, the electron affinity of the rubber is higher than that of the nylon. Under tensile force, the inner rubber fiber and the helical surrounding triboelectric layers structure are stretched, in which the surface of the PTFE and nylon fiber will be closely in contact with the rubber surface, and the electrons will transfer from rubber to PTFE on the interface of these two materials, while the electrons transfer from nylon to rubber on their interface. Therefore, the PTFE surface will become negatively charged, and the nylon surface will become positively charged eventually. Then, when the tensile force is removed, the inner rubber fiber and the helical surrounding triboelectric layers structure will shrink, in which the surface of the PTFE and nylon fibers will separate from the rubber surface, and the electron will transfer from the electrode of PTFE to the electrode of nylon to balance the electric potential. Therefore, the TENG generates electricity under this working cycle. Furthermore, COMSOL software is used to simulate the electric potential on the PTFE and the nylon fibers (Figure 1I), which is consistent with the mechanism description for electricity generation. To work steadily over time, the TENG should have high mechanical strength. Thus, the tensile strength of the TENG is studied, and the stress–strain curves of the four identical TENGs are collected in the tensile-loading test. As shown in Figure 1J, the TENG exhibits a strength of 25 MPa with a tension strain of more than 1700%, which effectively proved that the TENG has good tensile properties and great capacity to maintain long-term reliability.

**FIGURE 2:**
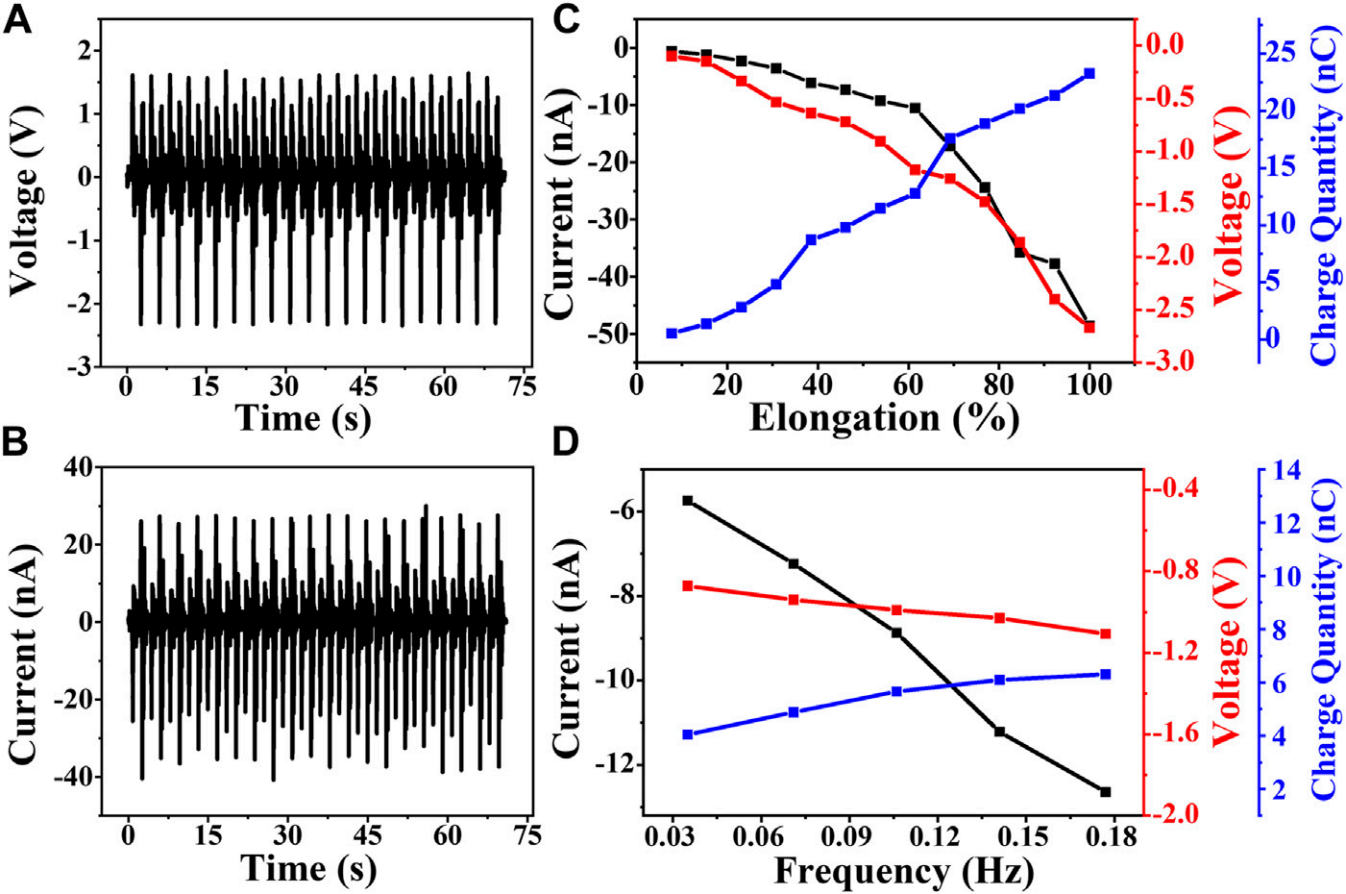
Output performance of the TENG under different conditions. **(A,B)** Voltage and current curves at 90% strain and 0.28 Hz. **(C)** Current, voltage, and charge quantity value with different strains at 0.28 Hz. **(D)** Current, voltage, and charge quantity value with different frequencies at 60% strain.

To test the output performance of the TENG, its one end is fixed at the measurement platform, and the other end is connected with the linear motor. When starting the linear motor to move back and forth at a working frequency of 0.28 Hz and displacement of 6 cm, the TENG will be stretched and loosened regularly, which generates an output voltage of 2.4 V and output current of 40.1 nA as shown in Figures 2A,B. In order to analyze the energy conversion ability toward the tensile force, the TENG is tested at a stretching frequency of 0.28 Hz. At the same time, the displacement of the TENG is adjusted to stretch the TENG to different strain lengths. As shown in Figure 2C, it can be found that under the tensile force of the linear motor, the TENG is persistently stretched. Also, when the strain increases from 10 to 100%, the current raises from −1 nA to −49 nA, the voltage raises from −0.11 V to −2.67 V, and the charge quantity raises from 0.9 to 23.3 nC, which can be attributed by the enhancement of an effective triboelectric effect. Also, when the strain increases from 10 to 60%, the output signals increase quite slowly. Further increasing the strain from 60 to 100%, the output signals increase by a greater margin, which can be attributed to a stronger triboelectric effect in a closer contact under larger strain. The tension–relaxation frequency may make a great effect on the output performance of the TENG, so we adjust the working parameters of the linear motor to stretch and release the TENG under different frequencies with a fixed tensile strain of 60%. As displayed in Figure 2D, when the tension–relaxation frequency increases from 0.035 to 0.177 Hz, the current raises from −5.8 nA to −12.6 nA due to the increase of the triboelectric charge separation rate. The voltage and the charge quantity raise quite slowly, which is mainly determined by the structure and materials of the TENG.

**FIGURE 3:**
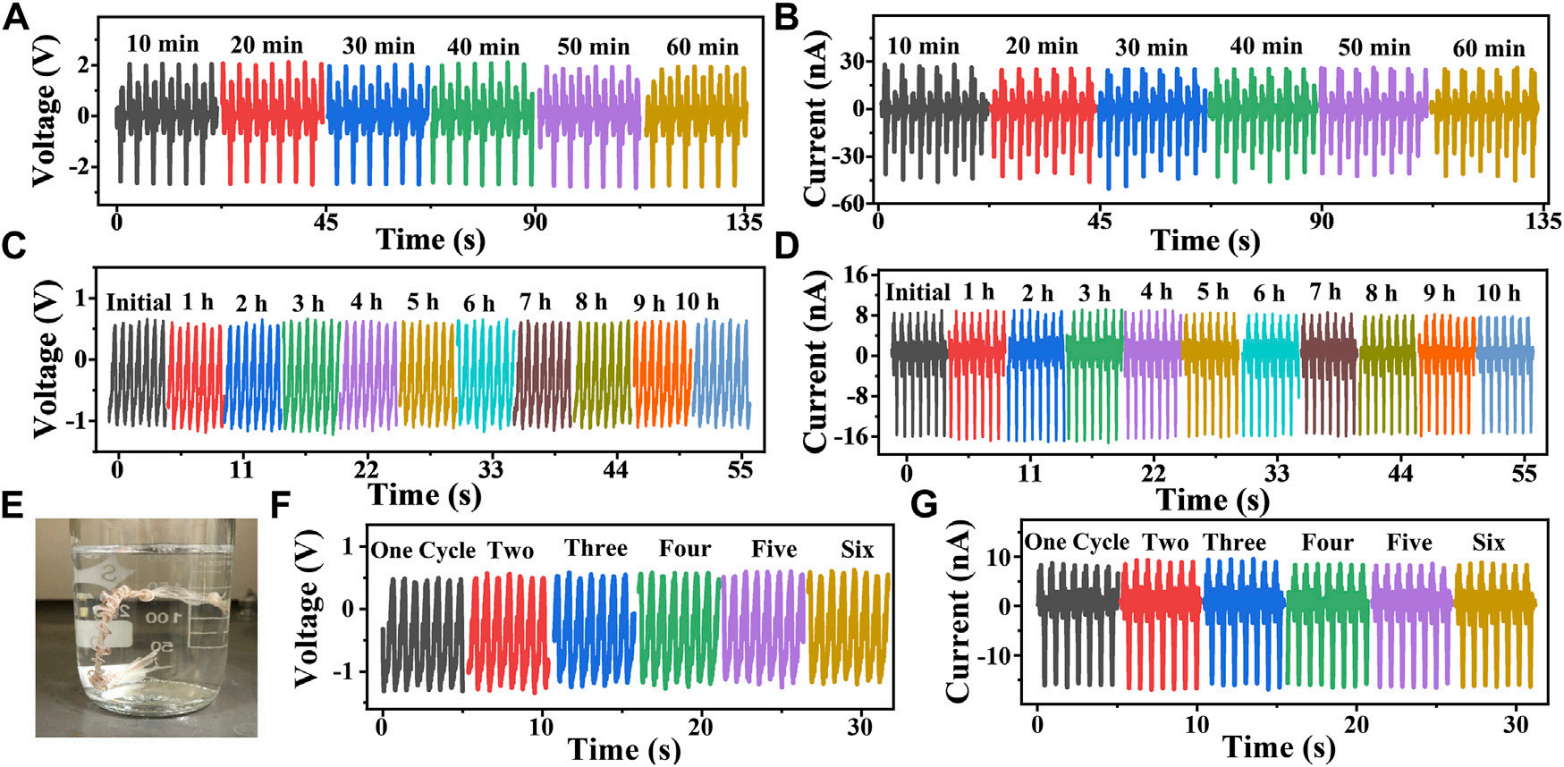
Mechanical endurance of the TENG. **(A,B)** Voltage and current curves measured at 90% strain and 0.28 Hz after holding the TENG stretching at 90% strain for different time periods. **(C,D)** Voltage and current curves measured with the TENG that was kept working at 70% strain and 0.14 Hz for 10 h. **(E)** Photograph of the TENG while washing. **(F–G)** Voltage and current curves measured at 70% strain and 0.14 Hz after washing the TENG for different cycles.

Mechanical endurance is very important for the supplication of the TENG, so we conducted three groups of experiments. At first, the TENG is stretched to 90% strain and then kept for a certain time (10, 20, 30, 40, 50, and 60 min). When the designed time is up, the TENG starts to work in loosing and stretching at 90% strain and 0.28 Hz driven by the linear motor. The voltage and current curves corresponding to different time periods in the 90% strain stretching state are measured and demonstrated in Figures 3A,B, which shows good resistance to mechanical tensile loading. Then, the TENG is continuously driven by the linear motor at 70% strain and 0.14 Hz for 10 h to measure if it is in a good condition. The voltage and current curves are measured every hour and displayed in Figures 3C,D, in which negligible attenuation is found and exhibits a highly stable working performance. As shown in Figure 3E, the TENG is immersed into the water, and the glass container is placed in a magnetic stirring apparatus after adding into a magneton. In this way, the TENG can be well washed. After washing and drying, the working performance of the TENG is also measured. Figures 3F,G show the voltage and current curves measured at 70% strain and 0.14 Hz after washing for six cycles. We can find that there is no reduction in the output performance after each washing, thus displaying good washing durability.

**FIGURE 4:**
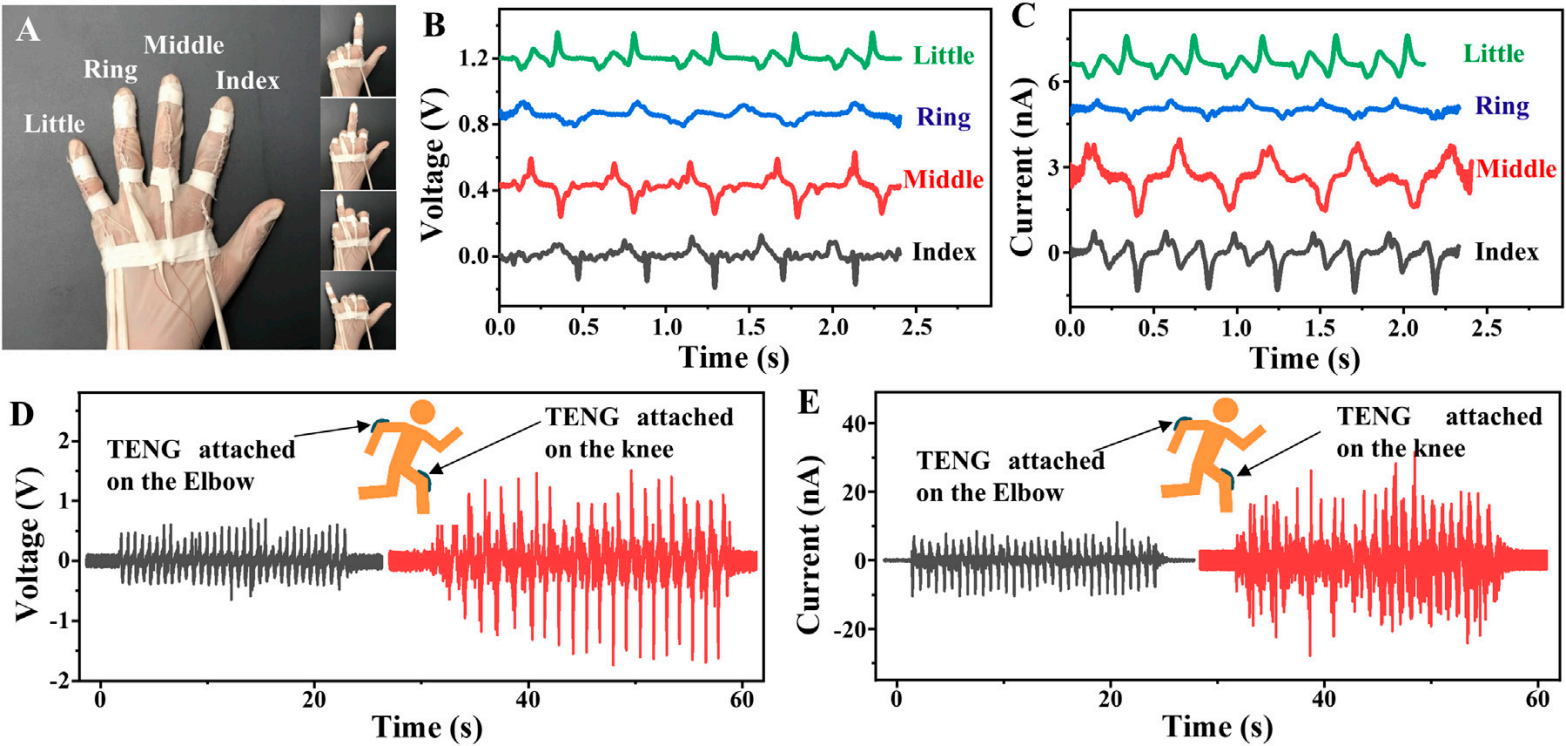
Sensing features to the human-body motions. **(A)** Photograph of a hand with four TENGs fixed on its four fingers from the index finger to the little finger. **(B,C)** Voltage and current curves of the four TENGs fixed on the four fingers. **(D,E)** Voltage and current curves of the TENG fixed on the elbow and knee.

As can be seen from the aforementioned experimental results, the TENG possesses good mechanical properties, especially tensile properties. Therefore, the TENG can be used to monitor hand finger movements. As shown in Figure 4A, four TENGs are attached to the index finger, middle finger, ring finger, and little finger, respectively. Every time the finger bends and stretches, the TENG fixed on it is also bent and stretched. The corresponding voltage and current signals generated by the four TENGs are detected and collected by bending these four fingers in turn from the index to the little finger as shown in Figures 4B,C. When each of the middle three fingers (index, middle, and ring fingers) is in the process of bending and straightening, the adjacent two fingers on both sides also follow slight reactions, resulting in a quite wide baseline. But the motion of the designated finger is rather stronger, so the output signals can be clearly distinguished in the real-time voltage and current signals, indicating promising applications in self-powered smart gesture recognition. Furthermore, its application in monitoring human body motions is achieved by attaching the three TENGs to the elbow and the knee. When people’s arms and legs bend, the TENGs are stretched, and electrical signals can be collected. As shown in Figures 4D,E (the left), there is one up and one down output peak every time the arm bends up to an angle of about 90°. At the same time, the output voltage and current reach 0.6 V and 10.6 nA, respectively. As for the TENG attached to the knee, it also responds well to the leg activity, and each time the leg bends backward to about 90° an output voltage of 1.7 V and current of 25.0 nA can be generated as demonstrated in Figures 4D,E (the right). This result proves that this TENG has great ability in converting human body motion into electrical energy as well as monitoring the human activities.

**FIGURE 5:**
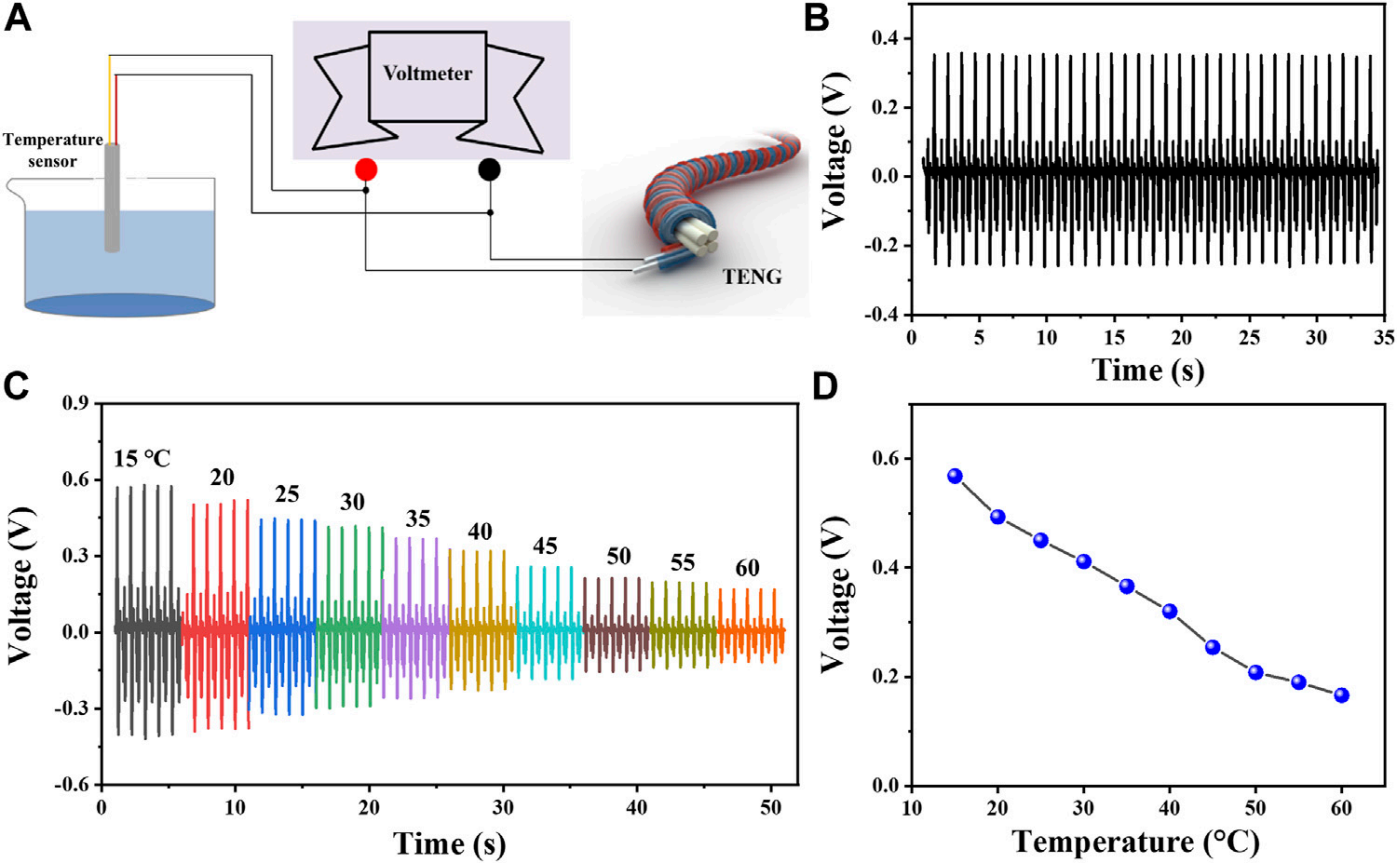
TENG-based self-powered temperature sensor. **(A)** Schematic diagram of the connecting circuit. **(B)** Stable voltage curves of the self-powered temperature sensor system working at 36.5°C. **(C,D)** Stable voltage of the self-powered temperature sensor at different temperatures.

People may work in different scenes, and the ambient temperature may vary greatly. Taking that into account, we assemble a self-powered temperature sensing system using this TENG and a temperature sensor. The connection circuit is depicted in Figure 5A. In this system, the TENG acts as the power supply for the temperature sensor. Also, when the temperature to be measured varies, the voltage between the two ends of the temperature sensor follows the change. At first, we tested the voltage on the temperature sensor at a temperature of 36.5°C. As shown in Figure 5B, it can be found that the voltage and current stay steady at 0.37 V under this condition. Then, to explore its detection range, the working condition is changed continuously from 15 to 60°C. Figures 5C,D give the voltage on the temperature sensor at different temperatures. We can find that the voltage signal decreases with the increase of the temperature, which can be attributed to the decrease of the internal resistance of the temperature sensor under higher conditions. It can be seen that this TENG, as a power supply unit, can effectively supply energy to the temperature sensor to ensure that it works properly without other power supplies. Furthermore, this test lays the foundation of this TENG in providing power to more sensors in the future.

## Conclusion

In summary, we have fabricated a fiber-based TENG with excellent flexibility and stretchability, which can be folded into different shapes and stretched to different lengths. Benefiting from the ingenious structure design composed of the elastic core fiber and helically surrounding triboelectric layers, it responds well to different strains and driven frequencies. Also, its stability and washability are measured to be good. Furthermore, it can be attached to the human body to monitor the finger, arm, and leg activities. At the same time, it can be used as the power supply to drive a temperature sensor in a self-powered sensing system. This work provides great application potential in multifunctional motion sensors and user-friendly health monitoring.

## Experimental Section

Measurement: a linear motor (LinMot E1100) is used to periodically drive the TENGs, and low-noise preamplifiers (SR570 and SR560) are used to measure the output voltage and current. PCI-6259 (National Instruments) is used for data collection. A software platform based on LabVIEW is used to realize real-time data acquisition and analysis. A strain gauge (Zhiqu, ZQ-990A) is used to test the stress–strain curve.

## Data Availability

The original contributions presented in the study are included in the article/Supplementary Material, further inquiries can be directed to the corresponding author.
